# Staged Custom, Intramedullary Antibiotic Spacers for Severe Segmental Bone Loss in Infected Total Hip Arthroplasty

**DOI:** 10.4061/2011/398954

**Published:** 2011-08-17

**Authors:** Atul F. Kamath, Okechukwu Anakwenze, Gwo-Chin Lee, Charles L. Nelson

**Affiliations:** ^1^Department of Orthopaedic Surgery, University of Pennsylvania, 3400 Spruce Street, 2 Silverstein, Philadelphia, PA 19104, USA; ^2^Department of Orthopaedic Surgery, Penn Presbyterian Medical Center, Cupp 1, 39th and Market Streets, Philadelphia, PA 19104, USA; ^3^Department of Orthopaedic Surgery, Geisinger Medical Center, Danville, PA 17822, USA

## Abstract

*Introduction*. Total hip arthroplasty (THA) infections with severe bone loss pose significant reconstructive challenges. We present our experience with two-stage hip reimplantation using an intramedullary, antibiotic-impregnated nail. *Methods*. Three patients with infected THA with severe proximal femoral bone loss (Mallory type IIIB or greater) were treated using a custom antibiotic spacer. Clinical outcomes and any complications were recorded. Average followup was 49 months from final reimplantation. *Results*. Mean age at spacer placement (stage 1) was 53 years. The mean Harris Hip Score at final followup was 80. Two patients had asymptomatic heterotopic ossification, and one patient had a 2 cm leg-length discrepancy. *Conclusions*. A custom intramedullary nail antibiotic spacer is a reliable option in the staged management of the infected THA with severe proximal femoral bone loss. Benefits of this technique include limb salvage with maintenance of leg length, soft tissue tension, and functional status.

## 1. Introduction

 Infection after total hip arthroplasty (THA) is a catastrophic complication that can cause significant pain and disability. Multiple failed reconstructive attempts, chronic infection, or trauma may result in significant bone loss, which further complicates reconstruction. Severe acetabular and proximal femoral bone loss can compromise limb stability and present significant challenges to the reconstructive hip surgeon. 

Two-stage reconstruction is the gold standard for treatment of periprosthetic joint infections [1–6]. Alternative management options include resection arthroplasty [7], multiple staged debridements, fusion, one-stage reconstruction [8–10], amputation, or chronic suppression antibiotic therapy. If adequate host bone is present, reconstruction with host bone can be done in the setting of one- [11] or two-stage [12] reconstruction. While two-stage reconstruction with antibiotic spacers may be successful in eradicating infection in up to 95% of patients [13–15], traditional reconstructive techniques fall short in the face of segmental bone loss or host factors that preclude use of host bone for reconstruction [16]. 

Custom devices substituting for severe segmental bone loss after infected THA provide an opportunity at limb salvage, with maintenance of leg length, soft tissue tension, direct antibiotic elution into the tissue bed, and possibility for improved functional status/ambulatory capacity. However, the manufacturing of these custom prostheses can be costly and impractical. The purpose of this report is to present a technique and clinical results, at greater than four-year follow-up, for staged reconstruction of the infected THA with severe proximal femoral bone loss using a custom intramedullary antibiotic spacer. 

## 2. Methods

**Figure 1:**
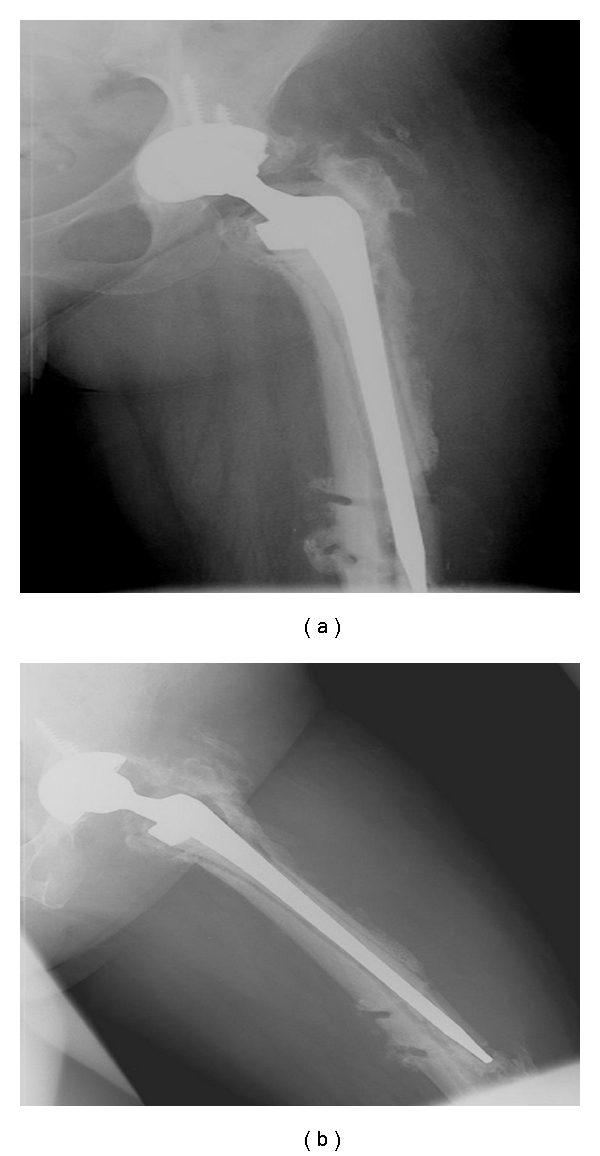
Anterior-posterior (a) and lateral (b) views after multiple prior revision attempts, demonstrating severe proximal femoral bone loss, component subsidence and compromise of the cement mantle, and perforation of the anterolateral femoral cortex of an infected THA.

We present three patients with infected hip prostheses managed with a custom antibiotic cement spacer fabricated out of a commercially available intramedullary nail. The patients underwent resection arthroplasties of an infected proximal femur replacement, a draining infected long-stem PROSTALAC, and long-stem THA, respectively. All patients had at least grade IIIB femoral defects according to the Mallory classification [17] or at least grade IIIA bone loss according to the system proposed by Della Valle and Paprosky [18] (Figures 1(a) and 1(b)). There were two males and one female, with an average age of 53 years (range, 43–61). All patients had multiple revisions prior to two-stage reconstruction: two patients had four prior revisions, and the other patient had three revision attempts. 

**Figure 2:**
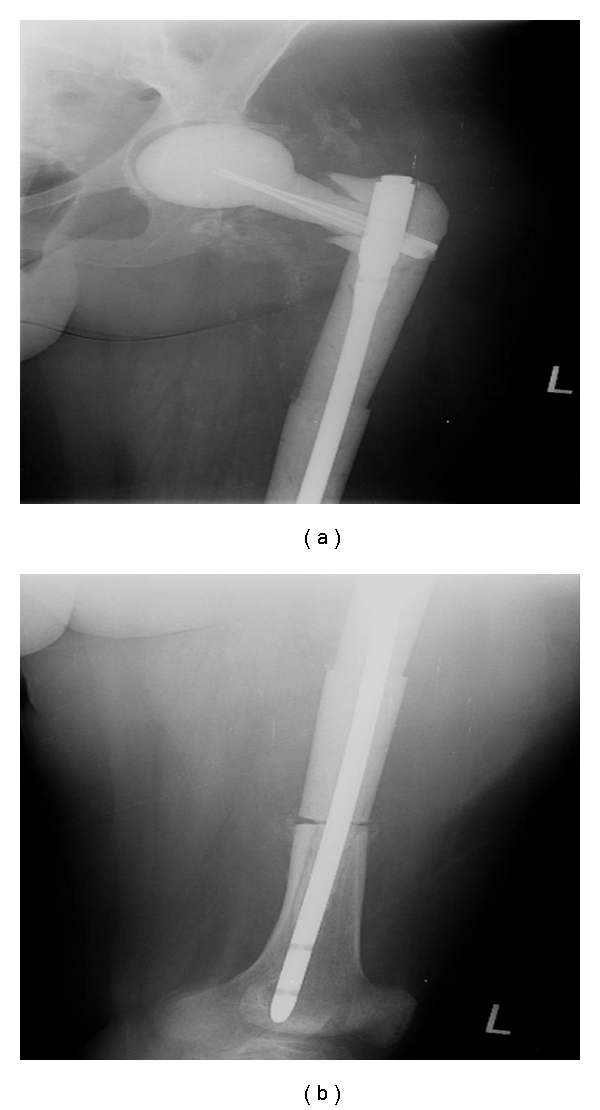
Anterior-posterior (a) and lateral (b) views after resection of infected THA and placement of a custom, antibiotic-impregnated cement intramedullary nail spacer.

**Figure 3:**
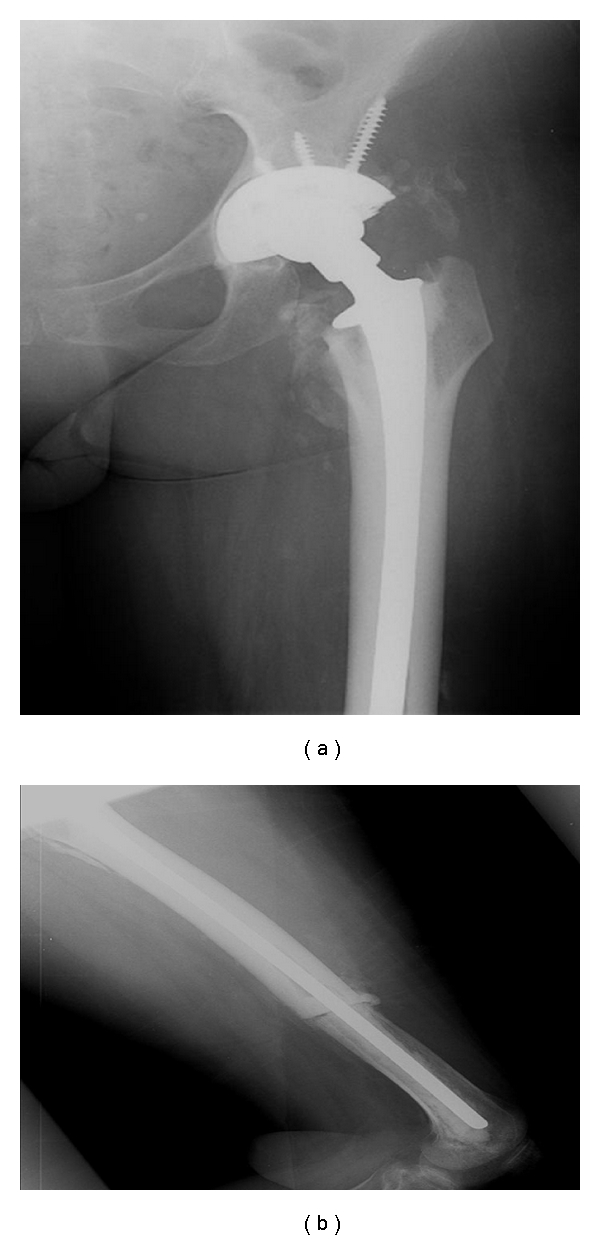
Anterior-posterior (a) and lateral (b) views of the femur after extraction of custom intramedullary nail spacer and definitive reconstruction with long-stem cemented THA prosthesis.

A cephalomedullary nail (Gamma 3, Stryker, Mahwah, NJ) was used with custom-molded antibiotic cement, following resection arthroplasty of the chronically infected THA (Figures 2(a) and 2(b)). The acetabular portion of the spacer mold was fashioned with the use of a bulb syringe to serve as a template. All procedures from index THA to final reimplantation were reviewed. The type and course of antibiotic regimens, inflammatory markers, clinical and functional outcomes, range of motion, and any complications were recorded. Radiographs were reviewed for any evidence of component loosening, subsidence, or fracture (Figures 3(a) and 3(b)). Following resection arthroplasty, all patients received culture-specific antibiotics for an average of 15 weeks (range, 6–28). 

## 3. Case Study

A 42-year-old man presented to our clinic for tertiary care consultation. He had undergone a THA seven years earlier for posttraumatic arthritis. His past medical history was significant for hepatitis C infection requiring liver transplantation in 1999, now maintained on cyclosporine immunosuppression. The index arthroplasty operation had been complicated by early loosening, necessitating revision surgery performed in his home country of Puerto Rico. On presentation, he complained of groin pain with hip internal rotation and thigh pain. Radiographs revealed a hybrid total hip implant with evidence of circumferential loosening about the femoral stem and proximal femoral osteopenia. 

The patient underwent revision of both acetabular and femoral components with trochanteric plate stabilization and impaction grafting. On initial postoperative visit, the femoral stem was noted to have perforated the thinned femoral cortex. The decision was made to revise his femur with removal of the implant and plate fixation of the femur with a long distal femoral plate to allow support at the fracture site. Unfortunately, he suffered a fracture at the point of high stress between the trochanteric plate and the distal femur plate.

At this point, eight months from initial presentation, he opted for a one-stage surgical salvage procedure. Considering the extent of bone loss and poor structural strength, a tumor style prosthesis was believed to provide the most predictable and reliable surgical option. Therefore a proximal femur replacement (Stryker, Mahwah, NJ, USA) was performed without complication.

The patient returned to his home country and did well for a year. However, he did not limit his activity after his last reconstruction and suffered two dislocation events. The first dislocation event was treated closed. However, the second incident required open reduction, which was performed in Puerto Rico. He then returned to our clinic, six months after his last dislocation and two years since reconstruction. He complained of hip pain and swelling about the scar region. A positron emission tomography scan was performed and revealed high uptake about the implant and surrounding tissues. Erythrocyte sedimentation rate and CRP were 35 mm/hr and 2.3 mg/dL, respectively. The clinical presentation, PET scan, and inflammatory markers were highly suggestive of periprosthetic infection. 

The patient elected for two-stage reimplantation and underwent initial resection arthroplasty of all components. A Gamma 3 nail coated with antibiotic impregnated cement was implanted as a spacer to provide stability in the presence of significant bone loss. Five packs of cement were used in total, with 3.6 grams of tobramycin used per bag of cement. Intraoperative cultures grew *Staphylococcus epidermidis*, and he was placed on intravenous vancomycin. The patient remained on intravenous antibiotic therapy for nine weeks, with serial labs and clinical examinations. Twelve weeks after spacer implantation, he underwent a second-stage reconstruction with a proximal femoral replacement with a constrained acetabular prosthesis (Zimmer, Warsaw, IN, USA). Final intraoperative cultures and frozen sections were negative.

At latest followup, 38 months after second-stage reconstruction, he continued to do well. He was able to walk comfortably with a cane and was able to sit comfortably for over an hour. Hip flexion was 100 degrees, extension to 10 degrees, abduction to 40 degrees, adduction to 40 degrees, internal rotation to 20 degrees, and external rotation to 60 degrees. The latest Harris Hip Score was 77. Radiographs at final followup revealed a well-fixed prosthesis with no evidence of loosening. 

## 4. Surgical Technique

Preoperative templating is essential to anticipate areas of bone loss and to prepare for type of reconstructive implant required. The hip is approached via a standard posterolateral approach to the hip. An extended trochanteric osteotomy can be performed in order to facilitate removal of the infected femoral component and/or infected cement. 

In cases in which acetabular bone stock is sufficient, the use of a cephalomedullary nail coated with antibiotic cement is an effective way to manage patients with severe proximal femoral bone loss. An intramedullary nail is used to form the core of the spacer. Preoperative templating and traction aided leg-length measurements are used to determine the length of the intramedullary nail required to achieve both axial and rotational stability. The acetabular size is estimated from the explanted hardware and intraoperative assessment following removal of all necrotic and nonviable bone. The use of hemiarthroplasty head sizers can be helpful in sizing and to provide a template for fabrication of the antibiotic cement acetabular ball head. Polymethylmethacrylate cement is hand-mixed with high dose concentrations of vancomycin and tobramycin (e.g., 3.6 grams of tobramycin per bag of cement), and the nail is encapsulated with cement in a custom-molded fashion. Once the cement is sufficiently doughy, it is molded around the nail to approximate the shape of resected bone and explanted prosthesis. The custom spacer is inserted into the canal when the cement is firm (but not hard), and the construct is reduced into the acetabulum. The limb is held at length and in the appropriate rotation during the final cement curing process. Usually, it is not necessary to place distal interlocking screws through the nail, however if rotational stability cannot be achieved, it is an option, but later concern for creation of a stress riser must be considered. Hardware placement is confirmed with intraoperative radiographs.

The hips in this study were reconstructed using the following implants: one proximal femoral replacement prosthesis with a constrained acetabular component (Zimmer, Warsaw, IN, USA), one proximal femoral replacement prosthesis with a constrained acetabular component (Stryker, Mahwah, NJ, USA), and one modular LINK cemented femoral prosthesis (Waldemar Link, Hamburg, Germany). Tissue cultures were obtained at time of stage 2 reimplantation, and patients were maintained on intravenous antibiotics pending final intraoperative culture results. No long-term suppressive antibiotic regimens were used. 

Postoperative rehabilitation included physical therapy starting postoperative day one. Partial weight bearing was allowed immediately, and ambulatory function and assistive aids were advanced as tolerated. Abduction bracing was used for prior history of dislocation. 

## 5. Results

The mean age at initial spacer placement (stage 1) was 53 years (range, 43 to 61 years). Average Charlson Co-Morbidity Index score was 2 (range, 1 to 3). Average BMI was 28 at initial presentation (range, 27–31). There were two smokers in the cohort. All patients had a traumatic etiology as a cause for the index THA. Two patients had a contralateral THA in place at initial presentation. Two patients had been diagnosed with osteopenia/osteoporosis over the course of the study period. There were no active Worker's Compensation claims during the study. One patient had a history of substance abuse.

The mean time between index THA and stage 1 procedure was 6.9 years (range, 15 to 129 months). All patients were ambulatory at time of initial presentation to our clinic but used some form of assistive device (e.g., cane, walker). Average ESR and CRP values prior to stage 1 procedure were 42 mm/hr and 5 mg/dL, respectively. Average estimated blood loss at stage 1 was 783 cc (range, 300–2000). At time of stage 1 explant/spacer placement, intraoperative cultures confirmed methicillin-resistant *Staphylococcus aureus*,* Staphylococcus epidermidis*, and vancomycin-resistant *Enterococcus*. The mean interval time between stage 1 procedure and final reimplantation (stage 2) was five months (range, 3–8). Average ESR and CRP prior to stage 2 procedures were 22 mm/hr and 1.0 mg/dL, respectively. There were no problems with extraction of the intramedullary spacer devices at stage 2. The average estimated blood loss at stage 2 was 1100 cc (range, 1000–1200). 

Complications included development of mild (Brooker Class II) heterotopic ossification in two patients [19]. There were no dislocations or fracture of the temporary spacers. There were no recurrent infections. One patient had a leg-length discrepancy of 2 cm at final evaluation. The mean Harris Hip Score was 80 (range, 77–84) at time of final evaluation. Two patients were free of assistive devices, while one patient used cane. 

## 6. Discussion

We report a series of patients with severe segmental femoral bone loss in the setting of periprosthetic hip infections managed with staged, custom-made antibiotic spacers. The amount of bone loss was too extensive for commercially available spacers and challenged traditional hip reconstruction algorithms, including previously described custom uses of antibiotic spacers [20–25]. Our primary research purpose was to define the clinical and functional outcomes, including eradication of infection, after two-stage reconstruction with custom-made spacers. The utility of these spacers as bridging procedures to ultimate reimplantation was of key interest.

The goals of two-stage reimplantation of infected THA are to eradicate infection [26], to provide local and systemic delivery of antibiotics at the infection site via the cement [27, 28], and to maintain the soft tissue envelope while awaiting reconstruction [29, 30]. Allowing postoperative mobility [31] is also essential to preventing complications associated with bedrest. When bone loss is minimal, standard spacers and commercially available devices are usually sufficient to accomplish such goals of revision THA [32–35]. However, when there is extensive bone loss in the proximal femur or acetabulum, options become limited.

The standard PROSTALAC articulated spacer, or other commercially available, prefabricated antibiotic-impregnated monopolar prostheses, like the Spacer-G (Tecres S.p.A, Verona, Italy) or antibiotic-loaded cement hemiarthroplasty (ANTILOCH) prosthesis, can be used effectively when there is minimal bone loss for standard reconstructions. However, these prostheses and techniques are not designed for extensive proximal femoral bone loss with little supporting host bone [36]. Severe segmental femoral or acetabular bone loss is not amenable to these reconstructive techniques. Commercially available or prefabricated/molded products may incur higher costs (in addition to cement costs) and may limit implant size choices intraoperatively [37]. 

Other techniques for antibiotic delivery include antibiotic beads. The use of antibiotic beads has not been shown to be superior to spacer prostheses implantation for restoration of functional mobility in two-stage reconstruction [13]. While there is a theoretical greater surface area for elution with beads [30], Tonegawa et al. found no long-term elution rate difference between beads and spacers with the use of gentamicin [38]. Limited use of antibiotic cement, such as antibiotic screws for infected intramedullary nailing [39], does not provide opportunity for adequate debridement, antibiotic delivery, and functional stability.

Kirschner-wire [30], Rush pin [40], or other modified custom-spacer techniques [25, 41] may not provide adequate strength, stability, or length to compensate for severe loss of bone stock. For example, periprosthetic fracture has been seen with the use of Kirschner-wire cemented spacers [30]. Hand-molded cement implants placed into areas of the proximal femoral metaphysis, even if reinforced with plates and/or screws, have been complicated by dislocations and component fracture [25]. Similar cement prosthesis fractures or dislocations have been seen in other studies [7, 32].

While Hsieh et al. successfully incorporated structural allograft in the second stage of reconstruction for massive femoral bone loss, there were two fractures and one dislocation of the custom-made interim antibiotic spacer [36]. Loty et al. and Rudelli et al. used allograft material in one-stage revisions for infected THA [42, 43], but this practice may have an unacceptably high infection rate, especially after prior failed reconstruction attempts [8]. Using antibiotic-impregnated allograft in one-stage revisions, Winkler et al. found a 92% infection-free rate at a mean of 4.4 years followup [44]. 

While antibiotic spacers have been demonstrated to be safe, there are potential systemic side effects [29, 45], which were not seen in our patient cohort. Moreover, there have been concerns with antibiotic resistance to cement-laden spacers *in vivo* [46]. With the emergence of multidrug resistant organisms and the projected increase in the absolute number of primary and revision arthroplasty procedures [30, 47], the number and complexity of periprosthetic infections will challenge future reconstruction algorithms. Other questions remain, such as the need, if any, and the duration of suppressive antibiotic treatment after final reconstruction stage [48].

There are limitations to this study. The relatively rare indications for use of the custom temporary spacers resulted in a small patient cohort. There was no control group to compare more traditional reconstructive or alternative custom-made spacer techniques. The surgical technique described may be applicable to tertiary care referral centers that regularly manage complex revision arthroplasty patients: there is a learning curve: the surgeon must be comfortable with the reconstructive options available and any potential perioperative complications, and the necessary support staff and resources must be readily available. 

To our knowledge, no study reports a series of full-length intramedullary nails with custom-molded antibiotic cement in the management of this complex reconstructive problem. One case report describes the use of an antibiotic-coated short-length cephalomedullary nail for treatment of an infected femoral intertrochanteric nonunion after compression hip screw placement [49]; no followup beyond 18 months is available for this patient. A case report has been presented on the use of a long-stem femoral prosthesis in the two-stage reconstruction of an infected THA with periprosthetic fracture [50]. 

## 7. Conclusions

We present a novel method of antibiotic delivery and limb stabilization during two-stage reconstruction of the infected THA. The technique, with greater than four years' followup, is a viable alternative to previously described treatment modalities.

Placement of custom devices substituting for severe segmental bone loss offers a reliable option in the staged management of the infected THA. Importantly, it provides an opportunity at limb salvage with maintenance of leg length, soft tissue tension, and functional status.
